# Text mining tools for extracting information about microbial biodiversity in food

**DOI:** 10.1016/j.fm.2018.04.011

**Published:** 2019-08

**Authors:** Estelle Chaix, Louise Deléger, Robert Bossy, Claire Nédellec

**Affiliations:** MaIAGE, INRA, Université Paris-Saclay, 78350 Jouy-en-Josas, France

**Keywords:** Microbial biodiversity, Text mining, Information extraction, Food spoilage

## Abstract

Information on food microbial diversity is scattered across millions of scientific papers. Researchers need tools to assist their bibliographic search in such large collections. Text mining and knowledge engineering methods are useful to automatically and efficiently find relevant information in Life Science. This work describes how the Alvis text mining platform has been applied to a large collection of PubMed abstracts of scientific papers in the food microbiology domain. The information targeted by our work is microorganisms, their habitats and phenotypes. Two knowledge resources, the NCBI taxonomy and the OntoBiotope ontology were used to detect this information in texts. The result of the text mining process was indexed and is presented through the AlvisIR Food on-line semantic search engine. In this paper, we also show through two illustrative examples the great potential of this new tool to assist in studies on ecological diversity and the origin of microbial presence in food.

## Highlights

• We present new text-mining tools to extract information in food microbiology.
• The results of the extraction are available in an on-line semantic search engine.
• Taxa, habitats, phenotypes and links between them can be queried in PubMed abstracts.
• Text-mining tools could assist to browse past and recent scientific literature.
• Two use-cases are presented: fruit microbiota and spore-forming bacteria in food.

## 1 Introduction

Food ecosystems are constrained by intrinsic factors (*e.g.* pH, salinity, water activity) and extrinsic factors (*e.g.* temperature, gas concentration or conservation process) (Doyle and Buchanan, 2012). There are more and more scientific studies that analyze, describe and explain microbial diversity in samples from specific food products with respect to these factors. Indeed, the generalization of omics technologies and analytical methods allows an easier exploration of different flora across similar food products. In particular, DNA-based technologies, such as high-throughput sequencing technologies, produce a large amount of information about microorganism species and strains identified from different environments (Bokulich et al., 2016).

It is now possible to study in depth the microbial composition of the flora, as well as the interactions that microorganisms develop with their environment and among themselves to identify major trends in food products. More generally, the production of biological data on microflora is increasingly easy. However collecting published information remains time-consuming, although it is highly valuable for the interpretation of experiments and for the design of further experiments. For instance, significant correlations between microbial species and their respective habitats and phenotypes are impossible to explore at a large scale. Scientific review papers are useful sources of information as they summarize the current state of knowledge on the microbial flora of given food products (*e.g.* microbiota in cheeses (Montel et al., 2014) or in raw meat (Doulgeraki et al., 2012)) and on the different types of food where a given organism is found (*e.g. Listeria monocytogenes* in European cheeses (Martinez-Rios and Dalgaard, 2018) or *Lactococcus piscium* in various food (Saraoui et al., 2016)). However, a collection of primary literature papers is highly difficult to summarize for three major reasons: the size of the corpus to be searched, the scattering of the information through several papers and databases (*e.g.* catalogs of collections of biological resource centers, sequence databases) and the lack of structure and standards shared between sources. New text mining and knowledge representation technologies that tackle these problems are emerging.

### 1.1 A large amount of scattered data

**Fig. 1:**
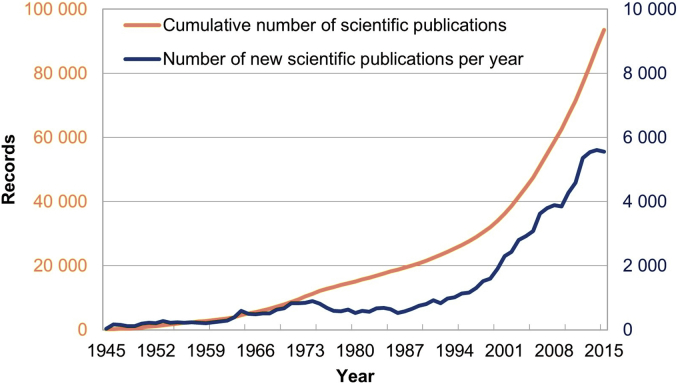
Increasing rate of publications in PubMed between 1945 and 2015 on the subject of food microbiology. The light orange curve represents the cumulative number of documents (left-hand scale). The dark blue curve represents the number of documents published per year (right-hand scale). See Material and Methods for further details. (For interpretation of the references to colour in this figure legend, the reader is referred to the Web version of this article.)

Fig. 1 illustrates the constant increase in publications related to food microbiology. The threshold of 1000 publications per year was exceeded in 1994, and there is a significant increase in publication number in 2005 (with more than 3000 articles per year). The amount of publications and the development of microbial identification techniques are correlated: in the 1990s some phenotypic methods were already cheap and easy to use. In the 2000s the release of genotypic methods and routine materials such as Next Generation Sequencing machine by Roche, and the GA sequencer from Solexa had a high impact on the identification of organisms and the study of the biological mechanisms involved in their adaptation and interaction (Escobar-Zepeda et al., 2015). Since 2013, the number of publications per year tops out at 5500.

A large amount of information about isolation sites and phenotypes is also scattered across several databases. Table 1 lists some of the major databases used for referencing either habitat or microbial phenotype information.

**Table 1:** Major databases with microbial habitat or phenotype information.

Database name	Institution	Relevant information entries
BacDive	DSMZ	24,150 “isolated from”
/	ATCC	18,000 “isolation”
GOLD	JGI	25,000 “isolation site”
GenBank	NCBI	60,000 “isolation source”

### 1.2 Unstructured information and lack of standards

Textual information is expressed in unstructured, natural language form at different levels of precision which makes it difficult to find. We illustrate language variability of microbe habitats by two examples on publications about cheese microflora. The relationship between the microbe and the cheese it has been isolated from, is expressed by “*Carnobacterium maltaromaticum* CPN, a strain isolated from unpasteurized milk Camembert cheese” in Hammi et al. (2016) and by “Only *Y. enterocolitica* was found to grow on the surfaces (outer and exposed) of Brie at all three storage temperatures” in Little and Knøchel (1994). In these examples, the habitat “cheese”, and more precisely “mould ripened cheese”, is denoted by the two different expressions “unpasteurized milk Camembert cheese” and “Brie”. The common bibliographic search engines use keyword queries that are too limited for taking into account such variability. A keyword query such as *cheese* fails to retrieve all of the relevant information. For instance, they miss documents where the proper name of the cheese (“Brie”) is used instead of the term “cheese”. Queries that include all cheese names are impractical to build and maintain. Moreover, keyword queries are not suitable for retrieving the relationships between the micro-organisms and isolation samples.

Knowledge resources such as controlled vocabularies, taxonomies and ontologies, bring a partial answer to the limitations of keyword-based search engines. Knowledge-based search engines extend simple string-matching with queries on general terms that do not depend on how they are expressed in the text. PubMed bibliographical database indexing with the MeSH thesaurus is a representative example.

Knowledge resources, in particular structured representation such as ontologies, also answer to information dispersion in various sources by providing a shared reference representation (Kelso et al., 2010). For example, many different databases share the Gene Ontology (Ashburner et al., 2000) for indexing gene properties.

### 1.3 Text mining for bibliographical research

Knowledge resources alone are insufficient to capture all the language variations. Text mining technologies combine knowledge resources, linguistic analysis, and machine learning to deal with language variations. Furthermore text mining tools can extract terms from text, but also relationships between terms. Text mining tools and methods have been used to analyze publications in several domains, especially in the biomedical domain (see examples in the review of Fleuren and Alkema (2015)).

Fine-grained information extraction achieves high performances in Life Science (Kim et al., 2011). The need for text mining in the microbiological field was identified more than a decade ago (Bessières et al., 2006), which we confirmed with a recent needs analysis, targeting food microbiologists (Chaix et al., 2017). The pioneering work on EnvDB database (Pignatelli et al., 2009) aimed to link GenBank sequences of microbes to biotope mentions in scientific papers. However, EnvDB was affected by the incompleteness of the GenBank isolation source field, the low number of related bibliographic references, the limited results of the text mining extraction method and the small size of its habitat classification. The few text mining projects applied to microbiology focus on biomedical aspects of the field. For example, the study on document classification related to type IV secretion systems bacteria (Ananiadou et al., 2011), and the application on bacterial enteropathogens (Zaremba et al., 2009) (no longer on-line). Their focus is mainly on gene detection in pathogenic microbes. MicroPIE is an example of extraction of microbial phenotypes: the MicroPIE bioinformatics application uses text mining tools to classify sentences according to 42 microbial phenotypes (Mao et al., 2016).

More generally, information retrieval about microbes was boosted by the text mining competitions on gene regulation and biotopes (Bossy et al., 2011, Bossy et al., 2015; Deléger et al., 2016). These competitions promoted the development of efficient tools to detect entities of interest and relationships in microbiology literature, without focusing on a particular biotope.

As far as we know, the food domain has never been targeted as such by text mining research despite the importance of the domain. This work proposes text mining tools along this line, to extract information relevant to the food microbiology domain from the scientific literature. The results are indexed by an ontology and can be queried by a semantic search engine intended for researchers in food microbiology.

## 2 Material and methods

### 2.1 Strategy and resources

In this section, we describe the text mining approach that we designed to extract information about food microbiology from scientific documents. Text mining applications usually consist of four components: (1) the text mining methods themselves; (2) the formal definition of the type of information to be extracted; (3) the collection of relevant documents, *e.g.* scientific articles (referred to as corpus); and (4) structured knowledge resources (such as taxonomies, dictionaries or ontologies) that contribute to the detection of textual information and its normalization. Normalization consists of assigning a same category from the knowledge source to similar pieces of text to make text mining results more easily used and interoperable with other applications. We detail each of these points in the following subsections.

### 2.2 Information to be extracted

Information extraction consists in recognizing specific pieces of information that have been pre-defined. These pieces of information include entities (*i.e.* terms that are of particular interest to a domain) and relationships between these entities.

We consider here three types of entity: *microorganism taxa*, *habitats* and *phenotypes*; and two relationships: the “*Lives_in*” relation between a microorganism and its habitat(s) and the “*Exhibits*” relation between a microorganism and its phenotype(s).

### 2.3 Corpus

To build a corpus of documents related to the food microbiology domain, we selected all publicly available abstracts through the PubMed services of the NCBI. The PubMed bibliographic database is not only a relevant source for this domain, but references are also available for text and data mining and they can be freely re-distributed and copied. This right is necessary for the display of the context of the extracted information to the user. We expressed PubMed queries with MeSH thesaurus keywords in order to identify relevant abstracts from both the microbe and food domains. Table 2 gives the MeSH terms that we identified as relevant to these two fields. Column 1 lists the main microscopic organism taxa. Column 2 lists PubMed main food topics, including processing and packaging.

**Table 2:** MeSH terms and tree numbers used for the corpus selection.

Microbe		Food domain	
Alveolata	B01.043	Diet, Food and Nutrition	G07.203
Amoebozoa	B01.046	Food Analysis	E05.362
Nematoda	B01.050.500.500.294		J01.576.423.850.100
Choanoflagellata	B01.175	Food and Beverages	J02
Cryptophyta	B01.206	Food Industry	J01.576.423
Diplomonadida	B01.237	Food Microbiology	H01.158.273.540.274.332
Euglenozoa	B01.268		N06.850.601.500.249.300
Fungi	B01.300		N06.850.425.200
Haptophyta	B01.400		N06.850.460.400.300
Mesomycetozoea	B01.500	Food Packaging	J01.576.423.200.375
Oxymonadida	B01.625		J01.576.423.850.600
Parabasalidea	B01.630		J01.576.761.400
Glaucophyta	B01.650.232	Food Quality	J01.576.423.850.730
Chlorella	B01.650.940.150.469		N06.850.601
Prototheca	B01.650.940.150.634		
Volvocida	B01.650.940.150.925		
Volvox	B01.650.940.150.950		
Desmidiales	B01.650.940.800.150.200		
Retortamonadidae	B01.675		
Rhizaria	B01.680		
Stramenopiles	B01.750		
Crenarchaeota	B02.075		
Euryarchaeota	B02.200		
Korarchaeota	B02.500		
Nanoarchaeota	B02.600		
Bacteria	B03		
Viruses	B04		

The U.S National Library of Medicine (NLM) publishes a set of MEDLINE/PubMed citation records each year. We used the 2016 PubMed release to select relevant abstracts covering the period from 1945 to early 2016 as shown in Fig. 1. From this source, two corpora have been built: (1) the so-called *MicrobioPubmed* corpus, which is a selection of all abstracts indexed by Mesh terms related to microorganisms (2,333,943 abstracts); and (2) the so-called *Food* corpus, which is a sub-selection of the *MicrobioPubmed* corpus indexed by Mesh terms of the Food domain (101,072 abstracts).

We will update the two corpora with the next annual release at the end of 2017, and we will then update them periodically using daily updates provided by the NLM.^1^

### 2.4 Knowledge resources

In this work, we used two external knowledge resources, the NCBI taxonomy and the OntoBiotope ontology at two steps, first for the detection of the entities in the text by the text mining process and then for the indexing and retrieval of the entities by the end-users of the application. The resources have hierarchical structures so that the information retrieval queries can be expressed at different levels of generality depending on the needs, from the very specific (*e.g.* strain, given local specific cheese) to the very general (*e.g.* taxon order, food).

#### 2.4.1 Taxonomy

We use the taxonomy of the NCBI^2^ to detect mentions of microorganisms in the documents and assign them a reference taxon. NCBI taxonomy keeps track of synonyms and renaming, which is useful information for old bibliography search. NCBI taxonomy is also used as a reference to describe organisms in many databases: NCBI databases such as Sequence Read Archive (SRA) or GenBank, but also in European Nucleotide Archive (EMBL-EBI) or DNA Data Bank of Japan (DDBJ) (Federhen et al., 2014). Using the same taxonomy to index textual and biological information will make cross-reference easier.

#### 2.4.2 Ontologies

There have been very few attempts at microbial habitat standardization that yield either to very small and insufficient classifications, like the one of the American Type Culture Collection (ATCC) (Floyd et al., 2005) or the Genomes Online Database (GOLD) (Ivanova et al., 2010), to the notable exception of OntoBiotope ontology. We have developed the OntoBiotope ontology since 2010. It is publicly available on the Agroportal^3^ website, which is the major portal for ontologies in the agronomy domain. To our knowledge, it is the most complete resource on micro-organism habitats and phenotypes with 3,000 classes.

As an ontology, OntoBiotope represents domain knowledge in a formal, conceptualized and unified way (Gruber, 1993). The domain classes are formally defined and linked together by formal relations. The hierarchical relation links classes that are subtypes of each other. For instance, the three classes “*kefir*”, “*yogurt*” and “*sour milk*” are subclasses of the larger class “*fermented milk*”. This formally means that “Kefir *is a* fermented milk”. It has proved useful for information extraction (Papazian et al., 2012) and *Habitat* entity categorization in text mining challenges (Bossy et al., 2015).

The Habitat branch, called “OntoBiotope Habitat” in the following, includes a subtree dedicated to food products. We built it by reusing the FoodEx classification of European Food Safety Authority (EFSA, 2015), which we complemented by knowledge of microbiology and food domain experts. We chose FoodEx because of its attention to microbiological issues, including hazard and processing. The current version of the Food subtree in OntoBiotope consists in 801 classes at seven levels. The main branches reflect microbiology food research topics. The “*Commodity and primary derivative thereof*” subtree classify ingredients according to their origin (*e.g.* meat, milk, seafood, egg, honey). The “*Processed food*” subtree classifies food in 16 classes, *e.g.* canned, cooked, frozen, fermented. A specific branch is dedicated to animal food.

In addition to microbial habitats, we extended OntoBiotope with a second major branch dedicated to microbial phenotype, called “OntoBiotope Phenotype” (Nédellec et al., 2017). It classifies microbial phenotypes according to stress adaptability, including energy source, community behavior, host interaction, morphology, motility, metabolism and response to external conditions such as temperature (*psychophile*), pressure (*piezotolerant*), acidity (*alkaliphile*), or salinity (*extreme halophile*). The current version of OntoBiotope Phenotype contains 323 classes. Since we started building OntoBiotope, other work on phenotype has been published. The OMP ontology (Ontology of Microbial Phenotypes) (Chibucos et al., 2014) could have been relevant, but some useful phenotypes are missing such as those relative to obligate conditions (*e.g.* “*obligate piezophile*”). Furthermore, it is not fully well-suited for text mining, because the labels of the classes are different from the vocabulary used in papers and databases (*e.g.* “*mesophilic growth*” for *e.g.* “*mesophile*”).

### 2.5 Text mining methods

We used the Alvis text mining platform to design the information extraction pipeline (Ba and Bossy, 2016). The pipeline is composed of two steps: (1) entity recognition and normalization and (2) relation extraction. The first step identifies relevant terms in text and normalizes them according to selected knowledge resources, *i.e.* entities are assigned a specific entry from a given taxonomy or ontology. Then, relation extraction establishes links between identified entities.

#### 2.5.1 Detecting and normalizing entities

The Alvis pipeline extracts terms that denote microorganisms, habitats and phenotype entities using linguistic-based and rule-based text mining methods.

To detect mentions of microorganisms, it automatically finds matches between text strings and NCBI taxa (canonical names and synonyms). It applies rules to recognize variations of microorganism names, for example, the variants of *e.g.“Helicobacter pylori”*: “*H. pylori*”, “*H pylori*”, “*Hp*”. Recognized microorganism names are then assigned their NCBI TaxID.

For *Habitat* and *Phenotype* entity detection, we use a strategy that has shown to perform well in a similar task (Ratkovic et al., 2012). It involves a deeper linguistic analysis in two steps. First, the YaTeA term extractor extracts all terms, noun phrases and adjectival phrases from the text (Aubin and Hamon, 2006). Then, the ToMap method looks for matches between candidate terms and classes from the OntoBiotope ontology (Golik et al., 2011). Terms and class labels that have a similar internal syntactic structure are mapped and a similarity score is computed. ToMap then chooses the term-class pair with the highest similarity score. If a term cannot be mapped to a class then it is discarded, meaning that it is neither a habitat nor a phenotype. In addition to the core algorithm, dedicated heuristics handle ambiguous cases for the two types of entity, *Habitat* and *Phenotype*.

**Fig. 2:**
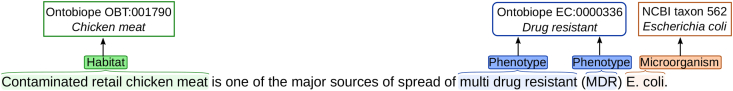
Example sentence with microorganism, habitat and phenotype entities.

Entity recognition and normalization is illustrated by the examples of Fig. 2. The highlighted portions of the sentence represent the entities: “*Contamined retail chicken meat*” as habitat, “*E. coli*” as microorganism, and “*multi drug resistant*” and “*MDR*” as phenotypes. The three square boxes at the top represent the class these entities have been assigned. The boxes show identifiers and class names from the knowledge resources. The “*Contamined retail chicken meat*” habitat entity has been assigned the more general class “*Chicken meat*” from the OntoBiotope ontology. The “*E. coli*” microorganism has been linked to the NCBI taxon “*Escherichia coli*”. Both phenotypes, “*multi drug resistant*” and “*MDR*”, are synonyms and have been matched to the OntoBiotope class “*Drug resistant*”.

#### 2.5.2 Extracting relations

The relation extraction method links recognized entities based on their proximity in the text (*i.e.* they must be part of the same sentence) and on linguistic cues called “trigger words” (Ratkovic et al., 2012). Trigger words are textual expressions that indicate a relationship between two entities. For instance, the expression “*isolated from*” usually shows a relation between a microorganism and its habitat.

The method also includes an anaphora detection component that links specific microorganism mentions to their anaphoric expressions. Anaphora are used to refer to entities previously mentioned in the text without repeating their name. For instance, authors may not repeat the name of a microorganism in the sentence describing its habitats and use a pronoun (*e.g.*, “*it*”) or a more generic term (*e.g.*, “*this microorganism*”) instead.

**Fig. 3:**
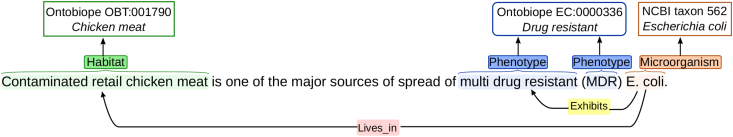
Example of relations between detected entities.

Fig. 3 shows the relationships between the identified entities from the example of Fig. 2. The “*E. coli*” microorganism is linked to the “*chicken meat habitat*” (by the *Lives_in* relation) and to the “*multi drug resistant*” phenotype (*Exhibits* relation).

## 3 Results

### 3.1 Descriptive statistics

We applied the Alvis pipeline to both *Food* and *MicrobioPubMed* corpora. It recognized almost 2 million entities in the *Food* corpus, among which 468,021 microbial taxa, 1,355,417 habitats, and 56,908 phenotypes (see Table 3). This accounts for 6.75% of the classes that were identified in the *MicrobioPubmed* corpus (respectively 5.59%, 7.32% and 5.80% of the *Taxa*, *Habitat* and *Phenotype*). In addition, more than 580,000 *Lives_in* and 19,000 *Exhibits* relations link these entities in the *Food* corpus (corresponding to 7.87% and 6.10% of the two kinds of relations in the *MicrobioPubMed* corpus). The proportion of extracted information is higher than the contribution of the *Food* corpus to the *MicrobioPubMed* corpus, which is 4.3%. The large number of extracted data unveils the amount of knowledge contained in the published documents, and the potential for discovery of additional knowledge.

**Table 3:** Descriptive statistics of text mining results.

	*MicrobioPubmed* corpus	*Food* corpus	
**Documents**	**2,333,943**	**101,072**	**(4.33%)**
**Entities**	**27,855,373**	**1,880,346**	**(6.75%)**
Habitat	18,514,216	1,355,417	(7.32%)
Microorganism	8,361,229	468,021	(5.59%)
Phenotype	979,928	56,908	(5.80%)
**Relation**	**7,777,691**	**606,717**	**(7.80%)**
Lives_in	7,465,205	587,645	(7.87%)
Exhibits	312,486	19,072	(6.10%)

### 3.2 Online search engine

#### 3.2.1 AlvisIR food search engine

The results of the text mining process on the *Food* corpus are made publicly available through the AlvisIR Food search engine (Fig. 5). It is accessible through a web browser to search for information about microorganism phenotypes and habitats at the location: http://bibliome.jouy.inra.fr/demo/food/alvisir/webapi/search.^4^

*Query interpretation.* The AlvisIR Food search engine is a semantic search engine. It interprets user query terms as taxonomy or ontology concepts, and expands each term with all synonyms and more specific concepts in their respective hierarchies. The result set contains all documents that have been annotated through the text mining process with these concepts.

*Relation query.* The AlvisIR Food search engine also features relational queries that allow the user to search for documents that contain specific relations between entities. This feature is unusual in a bibliographic search engine but more usual in database search. For instance, a user may search for microorganisms that exhibit a given phenotype (*e.g*. which *Staphylococcus* are anaerobic anaerobe?) or microorganisms that live in a given habitat (*e.g.* which *Acinetobacter* lives in fruits?). In order to be able to query these different aspects, queries can use special characters. The definition of the different possibilities are specified in the search engine, by clicking on the small icon “i” next to the search bar.

#### 3.2.2 Examples of semantic queries

Semantic search by AlvisIR Food handles different cases of synonymy. AlvisIR Food retrieves information for all taxa for a given class including renaming. For example the query *Petromyces* is interpreted as *Aspergillus*, and therefore returns all information related to this genus, since *Petromyces* has been renamed as *Aspergillus* (Samson, 2017), and the synonymy is recorded by the NCBI taxonomy. In addition to all synonymies in the NCBI taxonomy, Alvis also handles common typographic variations (*e.g.* abbreviation of the genus name).

Synonym management for habitats and phenotypes is more complex since no exhaustive list can be built in advance. The Alvis linguistic processing combined with the OntoBiotope ontology succeeds to capture many variations. For instance, it retrieves equivalent expressions for the sporulating phenotype: “spore-forming”, “endospore-forming” or “sporulation”.^5^

**Fig. 4:**
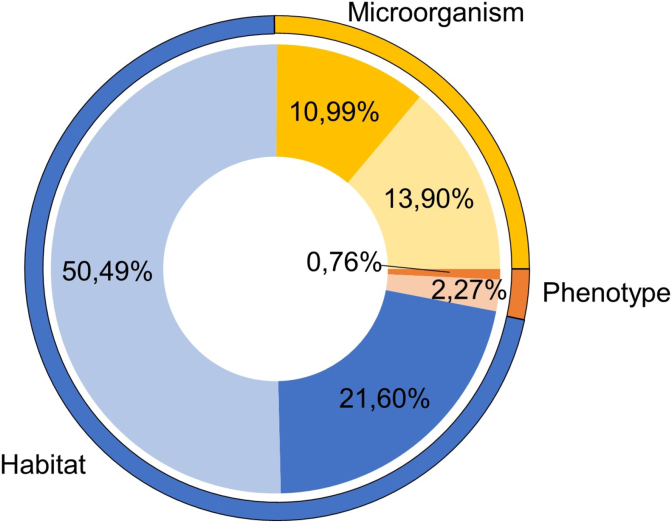
Quantity of entities (*Microorganisms*, *Habitats* and *Phenotypes*) predicted in the *MicrobioPubMed* corpus. Darker areas represent the proportion of entities that strictly matches a class name; lighter areas represent synonymous entities.

In order to assess the added value of the synonym expansion, we compared the number of entities predicted by Alvis with the number of entities that would have been retrieved by a simple string-matching method, as *e.g.*, Google Scholar does. 66% of entities that Alvis identified in the MicrobioPubMed corpus are different from the labels of taxon or ontology concepts, while only 34% are strict matches (as shown on Fig. 4.) Alvis then retrieves in average three times as many entities through synonymy expansion.

**Fig. 5:**
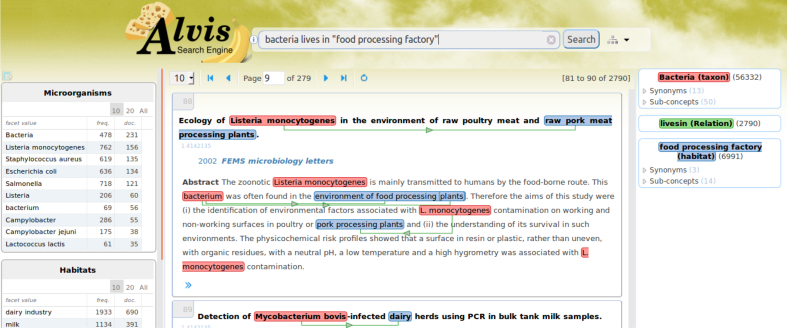
Screenshot of the AlvisIR Food search engine with the *bacteria lives in* “*food processing factory”* query. To the right are displayed synonyms of *food processing factory*, one of which is *food processing plant*.

Fig. 5 illustrates a relation query: *bacteria lives in* “*food processing factory”*.^6^ A hit abstract shows that a *Listeria monocytogenes* strain has been isolated in a “*raw pork meat processing plant*”. The green line represents the relation extracted between the bacterium and its habitat. The panel on the right displays the interpretation of the query, in particular synonyms and specializations of the food processing factory query term. This example illustrates the ability of the Alvis pipeline to detect and categorize new habitat terms (*i.e.* “*raw pork meat processing plant*”) and to link them to bacteria names.

Facets on the left side list microorganisms, habitats and phenotypes mentioned in the retrieved documents. They can be used to refine the query and target particular concepts, such as other bacteria isolated in the same biotope.

**Fig. 6:**
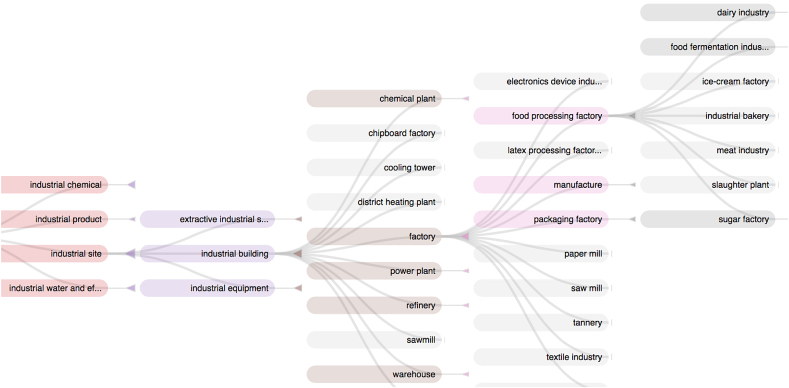
Screenshot of OntoBiotope browser with food factories displayed.

Writing a query may be difficult without knowing OntoBiotope terms. The interface provides a browsing facility that opens by clicking on the button next to the search bar. Fig. 6 shows the “*food processing factory*” branch from left to right. The user can build queries or refine previous queries by selecting or combining classes in this window.

## 4 Use of text mining results to investigate microbiological questions

The data extracted by text mining methods can be exploited in many ways including the investigation of complex research questions, which require the analysis of large amount of data. In this section, we look at two specific scientific questions to illustrate how text mining results can be used for food microbiology research:

• Which microorganisms have been isolated in fruits?
• Which microorganisms are known to be spore-forming and have been isolated in food products?

### 4.1 Microbiotope of fruits

Fruits can be eaten raw, and undergo few or no preservation processes. The various stages between production and consumption, and the external agents bringing germs (birds, transport, consumers touching the products *etc.*) are sources of microbial contamination (Heaton and Jones, 2008). Preservation processes, such as modified atmospheres or refrigeration may retain a flora in fresh fruits that can be harmful to the consumer; all the more so if the fruit is cut into pieces. The exogenous flora can contaminate the internal part of the fruit, whose intrinsic properties (*e.g.* water content and sugar resources) may cause microbes to develop (Oliveira et al., 2015).

Knowledge of the flora potentially contaminating a set of given fruits, is a valuable knowledge in many fruit processing applications, such as the design of new fruit desserts like ready-to-eat fruit salads. We propose here to show how text mining can contribute to the study of fruit microbial flora as a first step in the development of food products.

**Fig. 7:**
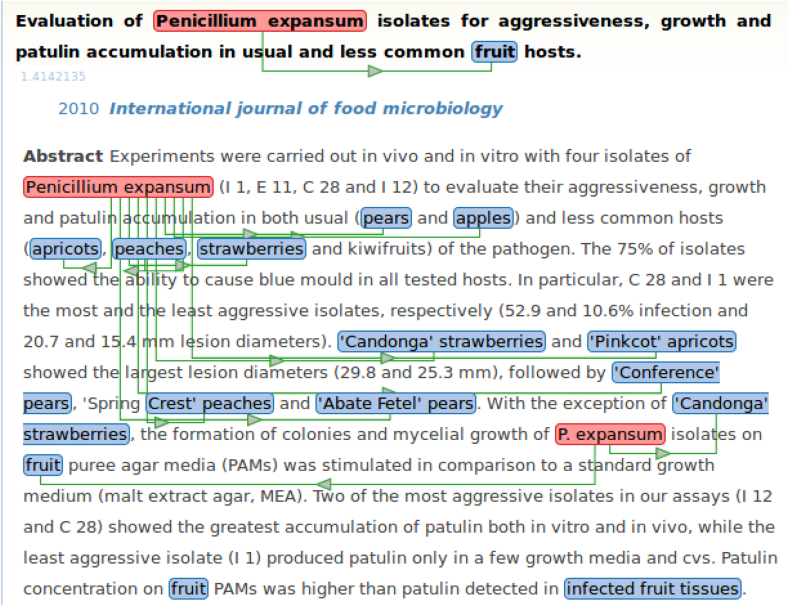
Screenshot of the AlvisIR Food search engine results to the query {taxon}*∼livesin fruit which means “which microorganisms live in fruit?”. Curly brackets are used to specify a query about the three main categories of entities, followed by the star * to indicate that they are all to be displayed (and mandatory): *i.e.* a query with all microorganisms is written as {taxon}*, a query with all habitats as {habitat}* and a query with all phenotypes as {phenotype}*. The presence of the tilde character ∼ (which is not mandatory) indicates that a query concerns a relation between entities (here, the *Lives_in* relation).

We queried the AlvisIR Food search engine to look for microbes living in fruits in the literature^7^. A query results are shown in Fig. 7. Table 4 shows the statistics of the query result: 10,546 relations are found between 993 unique microorganism classes (with unique NCBI TaxIDs) and 34 food fruit classes in 2,961 documents.

**Table 4:** “Microorganisms live in fruit” query statistics.

2961	documents
10,546	relations
993	unique taxa
34	fruit classes

**Fig. 8:**
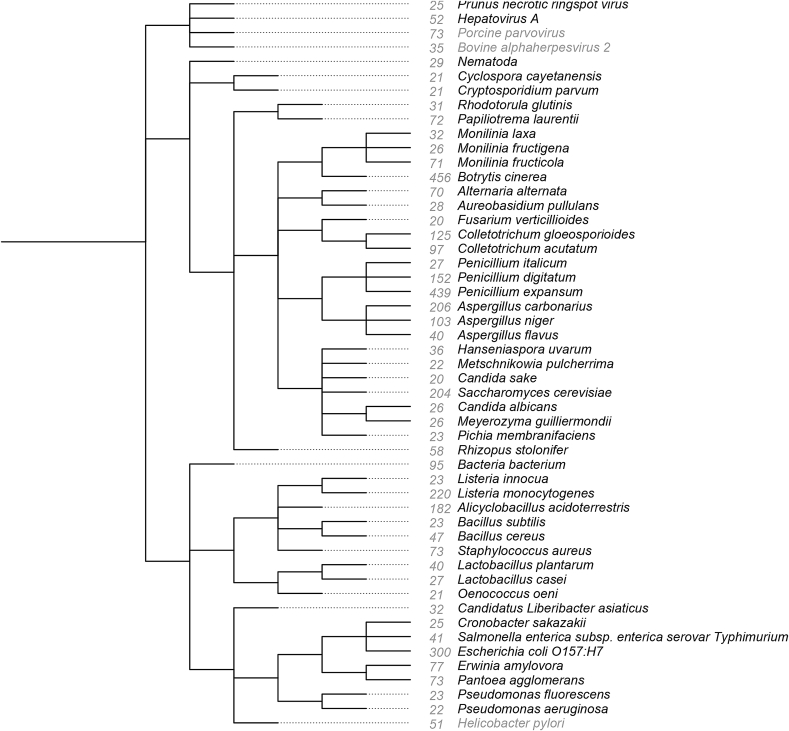
Phylogenetic tree of the main microorganisms, virus included, living in fruits as computed by the Alvis tool. The grey numbers are the number of relations extracted from documents. This figure was obtained using the TreeView software (Page, 1996) to view the PHYLIP format export from the NCBI CommonTree online tool, to which we gave the TaxIDs of all microorganisms present in food extracted by text mining processing. In grey, names of microorganisms wrongly recognized.

Fig. 8 shows the distribution of the microorganisms for which Alvis detected at least 20 *Lives_in* relations in fruits. We manually checked in documents that the relation was actually expressed at least once. Only three microorganisms, in grey in the figure, were wrongly recognized because of ambiguous acronyms. The main fungi found in fruits are *Saccharomyces cerevisiae*, *Botrytis cinerea*, *Penicillium expansum*, *Aspergillus carbonarius*, *Aspergillus niger*, *Penicillium digitatum*, *Colletotrichum gloeosporioides* and *Colletotrichum acutatum*; and the main bacteria are *Listeria monocytogenes*, *Escherichia coli* (and *E. coli* O157:H7), *Alicyclobacillus acidoterrestris*, *Salmonella enterica* and *Erwinia amylovora*. Our text mining tools also detected rare cases, such as *Povalibacter uvarum* isolated from a Japanese grape (a single relation was mentioned in Nogi et al. (2014)), *Weissella uvarum* found on wine grapes (a single relation mentioned in Nisiotou et al. (2014)), and *Prototheca wickerhamii* growing on bananas (three relations mentioned in Pore (1985)).

**Fig. 9:**
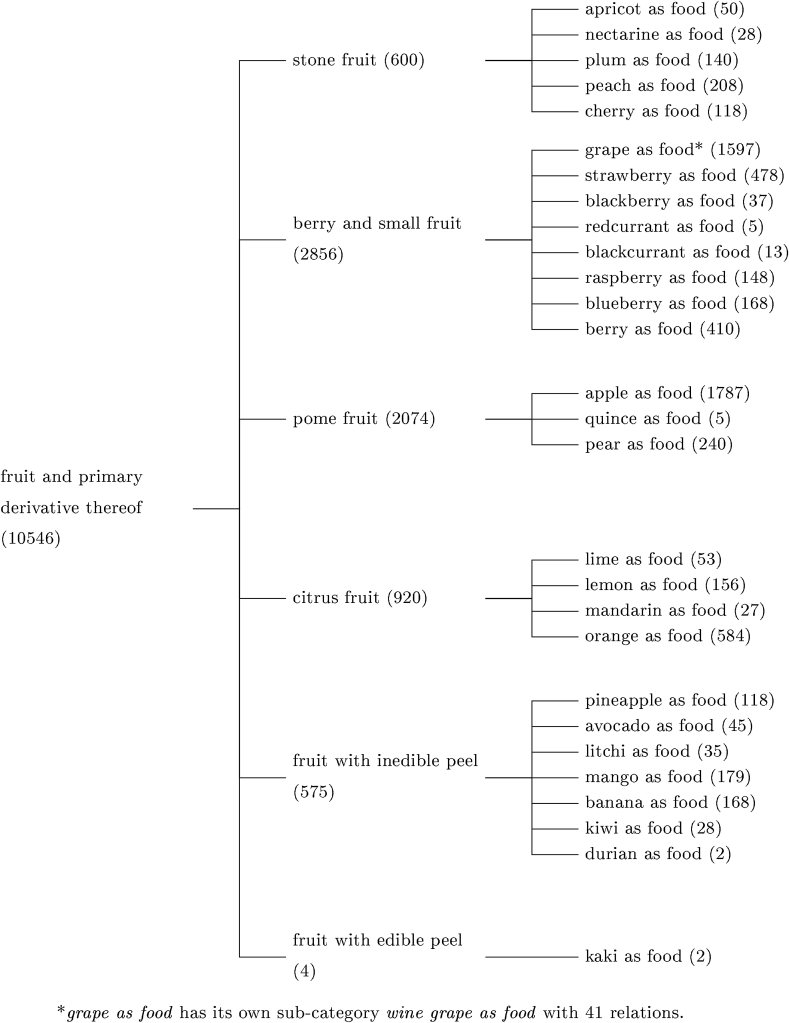
Number of relationships (between brackets) for each OntoBiotope subcategory for the {taxon}* livesin fruit query. The count in the highest classes cumulates the number of matches with that same class, as well as with its subclasses.

Fig. 9 shows the distribution of fruit classes mentioned in the query result set. The number of microbial studies reported in literature significantly varies depending on the fruit. For example, in the class “stone fruits”, there are 208 *Lives_in* relations from 128 abstracts for the peach, while only 20 relations from 18 abstracts for the nectarine. This case highlights the lack of information about the microbial biodiversity of some fruits.

Using text mining techniques, we can quickly search the bibliography. This makes it possible to estimate the potential risks that may exist, for example, in the case of designing a new fruit-based food product or replacing one fruit with another. In fact, text mining helps to identify the taxa of a fruit that could contaminate the final product. This knowledge can be refined by targeting the main microbes known to contaminate the different ingredients of a product (in our case study, the different fruits composing our fruit salad). This type of information from the literature has great potential, allowing us to think up the best way to preserve a fruit-based product according to the endogenous flora of each ingredient.

### 4.2 Sporulating microbes in food

In this section, we assess the potential of text mining for assisting the preparation of a review by comparing the information extracted automatically by text mining to the information of a review paper on a same subject. We selected spore-forming phenotype as a well-delineated subject of high interest for food processing.

#### 4.2.1 Biological objective

We focus on the identification of microorganisms that are capable of forming endospores, *i.e.* structures that allow them to resist to extreme conditions such as high temperatures, desiccation or high-pressure treatments. These resistant structures cause unwanted contaminations in the food industry, *e.g.* vegetable cannery (Durand et al., 2015) or milk product manufacture (Ranieri and Boor, 2009). Microbial spores that resist to treatments may contaminate food products, and by extension cause food poisoning (Postollec et al., 2012). Indeed, even though the spore is metabolically inactive, favorable environmental conditions may trigger its germination. Hygienic procedures and the various methods of food preservation, such as UV radiation, reduce the amount of spore-forming bacteria in the final food product (Brown, 2000). However, there are more and more cases where spores contaminate food and can develop when the conditions were theoretically not favorable, for example at low temperatures (Murphy et al., 1999) or after high heat treatments (Mtimet et al., 2016). Phenotypes of spore-forming bacteria are diverse, both in terms of their behavior towards oxygen and of their resistance to low or high temperatures. It is thus difficult to predict what type of bacterial taxa can be found in preserved food.

#### 4.2.2 Methodology

As a source of reference and for comparison, we use the work of Postollec et al. (2012) (and more precisely the first table), which lists spore forming bacteria in food products. 70 bacterial taxa were identified by the authors as such from manually browsing the literature or from their own expertise. Spore-forming bacteria occurred in various feed and food matrices such as silage, milk, fermented products and meat products.

**Fig. 10:**
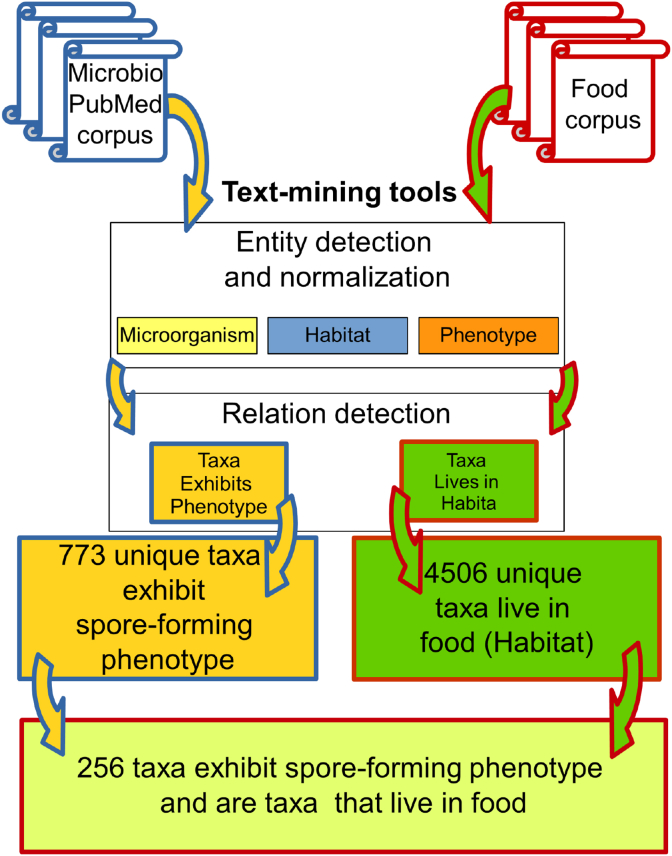
The text mining experiment workflow to answer the question “what taxa form spores and can live in food?”.

The text mining procedure is broken down into different stages as presented in Fig. 10. To recover taxa of organisms that are capable of forming spores, and that are also able to grow in food, we computed the intersection of the two lists: the spore-forming taxa and the ones living in food. This was possible because the formation of spores is a phenotype frequently mentioned in different articles. Alvis text mining tools have not been specifically tuned for this task so that this case study can serve as a basis to analyze errors and improve predictions.

The aim of this study is to measure (1) the amount of information that text mining retrieves compared to the review, (2) the amount of information that text mining misses, and (3) the amount of information retrieved that is not in the review and the reason for that. In order to qualify the errors of the text mining process, we will use the nomenclature of error analysis classification: the taxa that were incorrectly identified as spore forming in food are called false positives, and the taxa that were not found but should have been found are called false negatives.

#### 4.2.3 Prediction and comparison

The quantitative results of this experiment are presented in Table 5. The reference from Postollec et al. (2012) lists 70 taxa, among which 64 of specific ranks, strains and species. In the comparison with our findings we counted only once the lowest ranking taxa when two taxa of the same lineage were found (*e.g.* only *Clostridium perfringens* is counted in the pair *Clostridium* and *Clostridium perfringens*). The Alvis pipeline detects all kind of microbial taxa in the documents, regardless of the reign (bacteria or eukaryotes). The second part of Table 5 shows the results that are specific to bacteria to be compared to the review. The Alvis pipeline found 154 bacteria among which 37 are identical to the reference (58% of the reference). Alvis missed 27 taxa that were in the reference (*i.e.* 42% false negatives). 117 bacterial taxa were predicted, but were not in the reference. We have curated each of them by hand; they belong to two categories: 68 taxa were wrongly predicted (false positives) and 49 taxa were actually spore-forming. The Alvis contribution to the total number of spore-forming bacteria (70 plus 49) is then 41%, which represent a very significant increase of the state-of-the-art knowledge compared to the review.

**Table 5:** Quantitative results of the experiment on spore-forming taxa. The reference is the article of Postollec et al. (2012).

**All ranks of microorganisms**	
All taxas in Reference	70
Predicted by text mining	256
True positive (predicted and in Ref)	43/70
False Negative (not predicted but in Ref)	27/70
False Positive (predicted but wrong)	107/256
True positive, not in Ref	106/256
**Bacteria**	
(most specific ranks = taxonomic species or strain if available)	
Taxa (most specific ranks) in Reference	64
Predicted Bacteria (most specific ranks)	154
True positive (predicted and in Ref)	37/64
False Negative (not predicted but in Ref)	27/64
False Positive (predicted but wrong)	68/154
True positive, not in Ref	49/154

Manual analysis of false positive errors has shown that the wrong prediction of the *Exhibits* link between the taxon and the phenotype is the major source of error (69%). This preliminary work suggests efforts should focus on the improvement of the *Exhibits* relation extraction.

Other false positives are due either to the non-detection of the food entity or to the wrong detection of the relationship *Lives_in* (14%). In the same proportions, incorrect taxon categorization with the NCBI TaxID identifier (16%) induced a wrong *Exhibits* relation detection. This is due to ambiguous synonyms such as the mention of “strain MS1” in the work of Sankar et al. (2015) to refer to *Clostridium polynesiense*, while it is known as the synonym of *Alishewanella jeotgali* in the NCBI taxonomy.

Further examination of false negatives shows that 33% errors (9/27) are due to the lack of information in the corpus; there was no mention of the bacteria with food and/or spore-forming phenotype. 33% (9/27) are due to anaphora, a phenomenon known to be difficult to handle (*e.g.* microorganisms named only at the begin of paragraph). 19% (5/27) of the errors are due to missing “*Exhibits*” relations, and 15% (4/27) are due to missing habitats (*e.g.* no *gelatin* word in ontology). Some of these errors are trivial to correct, such as adding terms to the OntoBiotope ontology, or extending the corpus.

In order to further investigate the potential of text mining to complement existing sources of information we studied the content of the *BacDive* database (the DSMZ catalog of bacteria strains) with respect to the spore-forming phenotype. We focused on the 49 correct taxa that were found by text mining but absent of the reference. 30% are present in BacDive with the spore-forming phenotype. For instance *Geobacillus kaustophilus* is found in milk (Al-Tamimi et al., 2010), and sporulates (Al-Khalaf et al., 2012). On the other hand, 56% are present in BacDive but the phenotype spore-forming is not indicated. For instance *Paenibacillus humicus* is found in beer (Haakensen and Ziola, 2008) and is able to sporulate (Vaz-Moreira et al., 2007). Finally 14% are simply absent from BacDive, (*e.g. Coxiella burnetii* which is also found in milk (Hirai et al., 2012), and which has been shown to be capable of making spores (Marrie, 2003)).

These two comparisons illustrates how Alvis text mining tools can be efficiently used to complement existing reviews or databases by extracting relevant information from literature. We estimate the number of errors relatively small compared to the importance of the findings and the gain in time, including the curation time of the text mining results.

## 5 Conclusion

In this article, we proposed a new text mining approach that uses structured knowledge resources to extensively extract a very large amount of information about microorganism habitats and phenotypes from scientific literature in food microbiology. Our proposal addresses the lack of available structured information on this subject. We have detailed the text mining tools *i.e.* the Alvis platform that uses knowledge resources (*i.e.* OntoBiotope Habitat and Phenotype ontology) to deal with the high variability of the descriptions of the food microorganism properties. The resulting information is structured by relationships and hierarchies that one can efficiently search by using a semantic search engine, AlvisIR Food.

Through two use cases about a food product, “fruit”, and a phenotype, “spore-forming”, we have demonstrated the potential of Alvis text mining methods for fast literature review on biological questions by the analysis of millions of documents from the PubMed repository. Predicted results, with rapid manual curation, provide a quick overview of such questions that cannot be easily answered by manual bibliography review nor conventional search engines. Our future work will focus on the improvement of extraction of long-distance relations that are frequent in PubMed corpus. We will also update and extend the corpus with full-text papers by using the Alvis pipeline on the European text mining infrastructure OpenMinTeD. OpenMinTeD provides access for text mining tools to millions of documents from digital libraries. Finally, we will develop a database and an application programming interface to facilitate the use of this information by further bioinformatics processing. An example of such processing is checking the consistency of biological experiment results with the literature knowledge, *e.g.* strain identification in given samples, or hypothesis on the origin of a contamination. Assistance to curation and enrichment of existing databases is another obvious purpose to be developed. We believe that the range of potential uses of text mining in food microbiology is very wide.

## Footnotes

1 For more information, see the NLM website www.nlm.nih.gov.

2 NCBI taxonomy: https://www.ncbi.nlm.nih.gov/taxonomy.

3 OntoBiotope in Agroportal: http://agroportal.lirmm.fr/ontologies/ONTOBIOTOPE.

4 We recommend the following web browsers: Mozilla Firefox, Google Chrome or Internet Explorer. A detailed tutorial on the use of this engine is available at the following address: https://github.com/Bibliome/alvisir/wiki/How-to-use-Alvis-Search-Engine%3F.

5 See this search query example in the AlvisIR Food search engine http://bibliome.jouy.inra.fr/demo/food/alvisir/webapi/search?q=sporulating.

6 See the example at http://bibliome.jouy.inra.fr/demo/food/alvisir/webapi/search?q=bacteria+lives+in%22food+processing+factory%22+.

7 See this query on-line in the AlvisIR Food search engine http://bibliome.jouy.inra.fr/demo/food/alvisir/webapi/search?q=%7Btaxon%7D*+%7Elivesin+fruit.
